# A Quasi Time-Reversible Scheme Based on Density Matrix Extrapolation on the Grassmann Manifold for Born–Oppenheimer Molecular Dynamics

**DOI:** 10.1021/acs.jpclett.3c02098

**Published:** 2023-10-25

**Authors:** Federica Pes, Étienne Polack, Patrizia Mazzeo, Geneviève Dusson, Benjamin Stamm, Filippo Lipparini

**Affiliations:** † Dipartimento di Chimica e Chimica Industriale, Università di Pisa, Via G. Moruzzi 13, 56124 Pisa, Italy; ‡ CERMICS, École des Ponts and Inria Paris, 6 & 8 avenue Blaise Pascal, 77455 Marne-la-Valée, France; ¶ Laboratoire de Mathématiques de Besançon, UMR CNRS 6623, Université de Franche-Comté, 16 route de Gray, 25030 Besançon, France; § Institute of Applied Analysis and Numerical Simulation, University of Stuttgart, 70569 Stuttgart, Germany

## Abstract

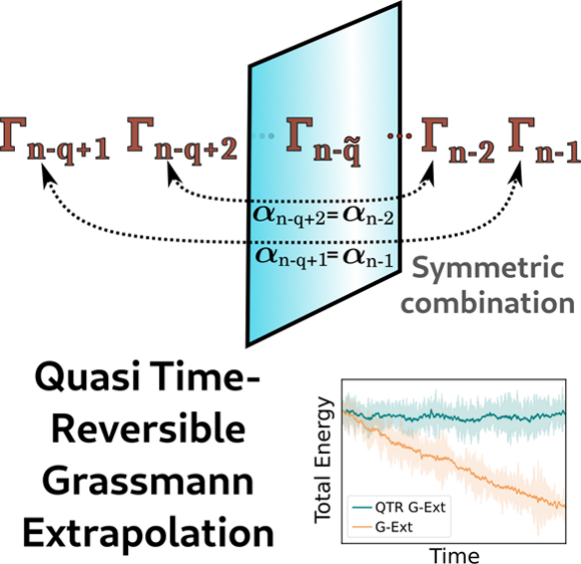

This Letter introduces the so-called Quasi Time-Reversible scheme based on Grassmann extrapolation (QTR G-Ext) of density matrices for an accurate calculation of initial guesses in Born–Oppenheimer Molecular Dynamics (BOMD) simulations. The method shows excellent results on four large molecular systems that are representative of real-life production applications, ranging from 21 to 94 atoms simulated with Kohn–Sham (KS) density functional theory surrounded with a classical environment with 6k to 16k atoms. Namely, it clearly reduces the number of self-consistent field iterations while at the same time achieving energy-conserving simulations, resulting in a considerable speed-up of BOMD simulations even when tight convergence of the KS equations is required.

Ab initio Born–Oppenheimer Molecular Dynamics (BOMD) is a very powerful and versatile tool to simulate molecular processes in which the quantum nature of the system is not negligible. Unfortunately, this comes at a high computational price, which stems from the necessity of solving quantum mechanical (QM) equations, typically Kohn–Sham Density Functional Theory (KS-DFT) equations, to compute the energy and forces at every time step. Such equations are nonlinear and are solved using a fixed-point iterative method known as Self-Consistent Field^1^ (SCF). Achieving SCF convergence typically requires, in a standard single-point run, up to 20 iterations, making the MD simulation very expensive, as in turn, the SCF has to be performed tens of thousands of times. Two main families of methods have been developed to address such a limitation. In extended Lagrangian methods, such as Car–Parrinello Molecular Dynamics (CPMD)^2^ or Atom-centered Density Matrix Propagation (ADMP),^3^ the electronic degrees of freedom are propagated, thus avoiding the need of solving the SCF problem. This requires one to endow the electronic degrees of freedom with a fictitious mass that needs to be small enough to keep the trajectory close to its Born–Oppenheimer counterpart. As a consequence, rather short time steps need to be used. A different strategy relies on developing extrapolation techniques^4−15^ for BOMD that allow one to converge the SCF in a limited number of iterations. In this work, we choose the second strategy, which is particularly effective for calculations using localized basis sets, e.g., Gaussian-type orbitals. The extrapolation techniques used in BOMD use converged solutions from previous MD steps to compute an accurate guess for the SCF, thus limiting the number of iterations required to achieve convergence. A significant contribution to this field was given by Niklasson and co-workers in 2006 with their work on the time-reversible extrapolation for Born–Oppenheimer Molecular Dynamics.^12^ The core concept involves generating a guess density matrix by combining the density matrices from previous steps in a symmetric and time-reversible manner. However, numerical applications showed that enforcing an *exact* time-reversibility can lead to errors accumulating in long-time simulations, thus spoiling the convergence properties of the algorithm in the long run. This led to the development of the Extended Lagrangian Born–Oppenheimer approach (XLBO) in 2008.^13−17^ In this particular case, the time-reversible extrapolation is augmented by the inclusion of a dissipative term, which serves to reduce the numerical fluctuations. XLBO can be seen as an intermediate strategy between Car–Parrinello like approaches and extrapolation techniques for BOMD, as it indeed propagates an auxiliary density matrix that can either be used directly in a CPMD spirit,^18,19^ possibly after refining the density using an approximate SCF solver, or be used as a guess for the SCF.^13^ Here, we focus on the latter approach.

In Niklasson’s XLBO scheme, the guess density is propagated over time, subject to a potential that forces it to be close to the converged density. The result is a guess density that is accurate enough to achieve reasonable SCF convergence (e.g., 10^–5^ RMS norm of the density matrix change) in as little as four iterations: Niklasson’s pioneering work has therefore been crucial in extending the applicability of BOMD. However, the XLBO method suffers from a few shortcomings. First, the guess density obtained with XLBO is not exactly idempotent,^13^ unless it is postprocessed using, e.g., McWeeny purification.^20,21^ Second, its performance degrades if a tightly converged SCF solution is required, as is the case when a post-Hartree–Fock method is used to compute the energy and forces (e.g., in a time-dependent DFT excited-state simulation).

Recently, we proposed a different strategy to compute a guess density by using linear extrapolation. This is nontrivial, because in general a linear combination of density matrices does not preserve idempotency or, in other words, density matrices belong to a differentiable manifold called the Grassmann manifold and not to a vector space. Our approach uses tools from differential geometry to map the Grassmann manifold onto its tangent space, which is a vector space. It then performs a linear extrapolation on the tangent space and then maps back the extrapolated density to the manifold. We named such a method Grassmann extrapolation (G-Ext) .^21,22^ G-Ext is an accurate and efficient strategy for ab initio MD simulations that has been shown to outperform XLBO, especially if a tight SCF convergence is required.^21^ It has been successfully adopted in the Pisa-group for both ground- and excited-state SCF-based BOMD simulations in a polarizable multiscale framework.^23−26^ Unfortunately, G-Ext suffers from a serious shortcoming. Numerical experiments have shown that the extrapolation introduces a bias causing a drift in the total energy for NVE simulations.^21^ Such an energy drift is modest in absolute terms (few kcal/mol in 10 ps to be compared with total energies of hundreds of thousands kcal/mol) but large if compared with the energetics of typical chemical processes. While using a tight convergence criterion for the SCF solves the problem,^21^ this is not an option for expensive, production simulations, thus limiting the gains introduced with the overall approach.

In this contribution, we not only address such a limitation by introducing a new strategy to perform the extrapolation but also further improve the performance of the method. We name the new strategy the Quasi Time-Reversible Grassmann extrapolation method (QTR G-Ext). This approach leverages the principles of differential geometry, similar to the previous method, but offers enhanced accuracy, improved performances, and excellent energy conservation properties. Given a  -dimensional atomic orbital (AO) basis, the SCF solves the following nonlinear eigenvalue problem, which consists of finding a matrix *C* and a diagonal matrix *E* such that
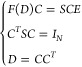
where  contains the  coefficients of the *N* occupied molecular orbitals,  is the density matrix,  is a diagonal matrix in which the entries are the energy levels, *F* denotes the DFT operator,  is the overlap matrix, and *I*_*N*_ denotes the identity matrix of order *N*.

We assume that the density matrix is orthogonal. In any case, it can be transformed into such matrix by considering the Löwdin factorization of the overlap matrix *S* and consequently the modified coefficient matrix . Then, the normalized density matrix  belongs to the manifold
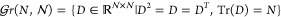
which is isomorphic to the so-called “Grassmann manifold”; therefore, we identify  by this name. From now on, we assume that the density matrix has been orthonormalized, and we denote it by *D*.

Since  is a differential manifold, given a point , there exists a tangent space , such that tangent vectors  can be associated with nearby points .

In MD, *t* → ***R***(*t*) represents the trajectory of the nuclei. The transformation of the electronic structure can be interpreted as a trajectory denoted by *t* → *D*_***R***(*t*)_ on the manifold. In order not to burden the notation, we simply indicate *D* in place of *D*_***R***(*t*)_. The objective is to determine a suitable approximation for the density matrix in the next step of the molecular dynamics trajectory by extrapolating the densities from previous steps. Since the tangent space  is a vector space, we approximate the density matrix on . In order to solve the extrapolation problem, we decompose the mapping ***R*** → *D* as a composition of several maps
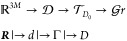
1where the first function ***R*** → *d* is a map from atomic positions to molecular descriptors. Here, as a descriptor, we use the Coulomb matrix^27^,
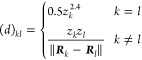
2where *N*_QM_ is the number of atoms treated quantum mechanically and *z*_*k*_ and ***R***_*k*_ denote the nuclear charge and the position of the *k*th atom, respectively. Note that other descriptors can also be considered. We will detail the crucial mapping of *d* → Γ below. The mapping of Γ → Exp(Γ) = *D* is the so-called Grassmann exponential which maps tangent vectors on  to , and it is a locally bijective function in a neighborhood of *D*_0_. Its inverse *D* →  Log(*D*) = Γ(*D*) is the Grassmann logarithm. These mappings are computed by means of singular value decomposition (SVD). For mathematical details, the interested reader is referred to refs (22, 28, and 29). In our method, during MD, we use a fixed reference point *D*_0_ to construct the tangent space .

Let *n* be the current time step of the MD. Given previous *q* snapshots Γ_*n*–*i*_ = Log(*D*_*n*–*i*_), for *i* = 1, ..., *q*, the approximation of the density matrix representative on the tangent space is written as
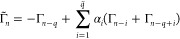
3where *q̃* = *q*/2 if *q* is even, while *q̃* = (*q* – 1)/2 if *q* is odd. We remark that, if in eq 3, the term Γ_*n*–*q*_ is substituted by , a “fully” time-reversible approach (instead of quasi time-reversible) is obtained. Numerical experiments with the fully time-reversible approach, which are reported in the Supporting Information (SI), showed good behavior for total energy conservation but, unfortunately, a strong increase in the number of performed SCF iterations. This is consistent with what has been observed by Niklasson and co-workers,^14^ who remark that exact time-reversibility under noisy conditions (e.g., not fully converged SCF) can lead to error accumulations and significantly worse SCF convergence.

The descriptors are involved in the computation of the coefficients  appearing in eq 3. Indeed, they are computed by solving the least-squares problem with Tikhonov regularization
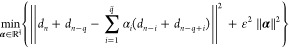
4where ∥·∥ denotes the -norm and ε > 0 is the regularization parameter. Since the Coulomb matrix (2) is symmetric, in the above formula, *d*_*j*_ represents the vectorized Coulomb matrix considering the lower triangle. In matrix form, it corresponds to solving the following least-squares problem
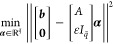
5where the vector ***b*** = *d*_*n*_ + *d*_*n*–*q*_ is padded with *q̃* zeroes,  is the matrix in which columns are defined as *A*_·,*i*_ = *d*_*n*–*i*_ + *d*_*n*–*q*+*i*_, and  is the identity matrix of order *q̃*. Then, the initial guess for the density matrix is obtained as the composition of the three maps in (1), where the second map *d* → Γ is given by (3). Note that if this second map denoted by *f* was linear, then the guess would be close to exact, namely
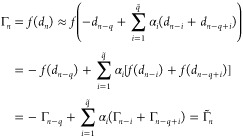
6After computing the coefficients α_*i*_ by solving (4) and the tangent vector  by eq 3, we obtain the sought guess density matrix for the SCF iterative method as .

The number *q* of density matrices taken at previous steps and the value of the regularization parameter ε are chosen in a heuristic manner: we computed the error  for different values of *q* and ε, specifically *q* = 3, 4, ..., 20 and ε = 0.001, 0.002, 0.005, 0.01, 0.02, 0.05, and we selected the combination (*q*, ε) corresponding to the minimal error. When the SCF convergence threshold is 10^–5^, we found that good values are *q* = 5 and ε = 0.005, while if it is fixed at 10^–7^, we found *q* = 4 and ε = 0.001, 0.002. Additional details on the selection of *q* and ε values can be found in Section S1. The computational cost to compute the extrapolation coefficients **α** is negligible compared to the time for a single MD step. Thanks to the symmetric property of the coefficients, the size of the system (5) is , and *q̃* is a small number (in our simulations *q̃* = 2, as *q* = 4 or *q* = 5).

The QTR G-Ext approach is tested on four different systems. The first system is dimethylaminobenzonitrile (DMABN) in methanol. The second system is 3-hydroxyflavone (3HF) in acetonitrile. The last two systems (OCP and AppA) are chromophores embedded in a biological matrix, namely, a carotenoid in the orange carotenoid protein (OCP) and a flavin in the AppA Blue-Light Using Flavin photoreceptor.^23,24,30^ Some information about the systems is reported in Table 1.

**Table 1:** Summary of the Systems’ Size: Number of QM atoms *N*_QM_, Number of MM Atoms *N*_MM_, Number of QM Basis Functions , Number of Occupied Orbitals *N*, and Size of Descriptors *N*_*d*_

system	*N*_QM_	*N*_MM_	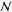	*N*	*N*_*d*_
DMABN	21	6843	185	39	234
3HF	28	15046	290	62	409
AppA	31	16449	309	67	468
OCP	94	6058	734	154	4468

KS-DFT has been adopted to describe the QM subsystem with the B3LYP hybrid functional^31^ and the 6-31G(d) Pople’s basis set.^32^ This is coupled with a polarizable description of the environment using the AMOEBA force field.^33^ For each system, we performed a QM/AMOEBA geometry optimization until a root-mean-square norm on the forces of 4 kcal/mol/Å was found, and finally, a 2 ps QM/AMOEBA NVT equilibration was performed to obtain the starting point of the simulations presented in this work.

All simulations have been performed using the Gaussian–Tinker interface.^34−37^ We implemented the QTR G-Ext extrapolation approach in Tinker.^38,39^

To assess the quality of the guess density obtained by the QTR G-Ext extrapolation, we performed 10 ps BOMD simulations with a 0.5 fs time step in the NVE ensemble, using the velocity Verlet integrator.^40^ All systems were tested with an SCF convergence threshold fixed to 10^–5^ and 10^–7^ with respect to the RMS variation of density. We compare our approach in terms of energy stability and number of iterations required to reach convergence with two other extrapolation schemes, which are the G-Ext scheme^21^
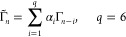
where α_*i*_ is computed by solving
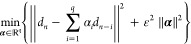
where ε = 0.01, and XLBO^13,14^
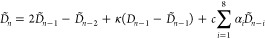
with fixed parameters κ = 1.86, *c* = 0.0016, and **α** = (−36, 99, −88, 11, 32, −25, 8, −1).

Figure 1 provides the plot of the total energy along the DMABN simulation with a 10^–5^ SCF convergence threshold. Despite the nonfully time-reversible formulation of our newly implemented approach, we observe a great improvement with respect to the G-Ext scheme. In particular, the results obtained with the QTR G-Ext method resemble the ones obtained the XLBO scheme. The same behavior is almost imperceptible when the SCF convergence is set to 10^–7^ (Figure 2), since the accumulation of errors that generates the energy drift when G-Ext is used is lower, so we can appreciate the same trend with all the extrapolation schemes. Analogous figures are reported in Section S2 for all tested systems. To better evaluate the energy stability, we consider the average short-time fluctuation (STF) of the energy, which is computed by getting the RMS of the energy fluctuation every 50 fs and averaging over the trajectory, and the long-time drift (LTD) for a long-time analysis, that is, the slope of the linear regression line of the energy. Tables 2 and 3 disclose STF and LTD for convergence thresholds of 10^–5^ and 10^–7^, respectively. QTR G-Ext, G-Ext, and XLBO show comparable STF, which is specific for the system and is related to the time step for the integration. On the other hand, the absolute value of LTD is in general higher for 10^–5^ simulations, in particular for G-Ext. We can state that the QTR G-Ext method solves the energy-drift issue of G-Ext, showing an LTD that is always similar to the XLBO one, suggesting again a good time-reversible behavior.

**Figure 1:**
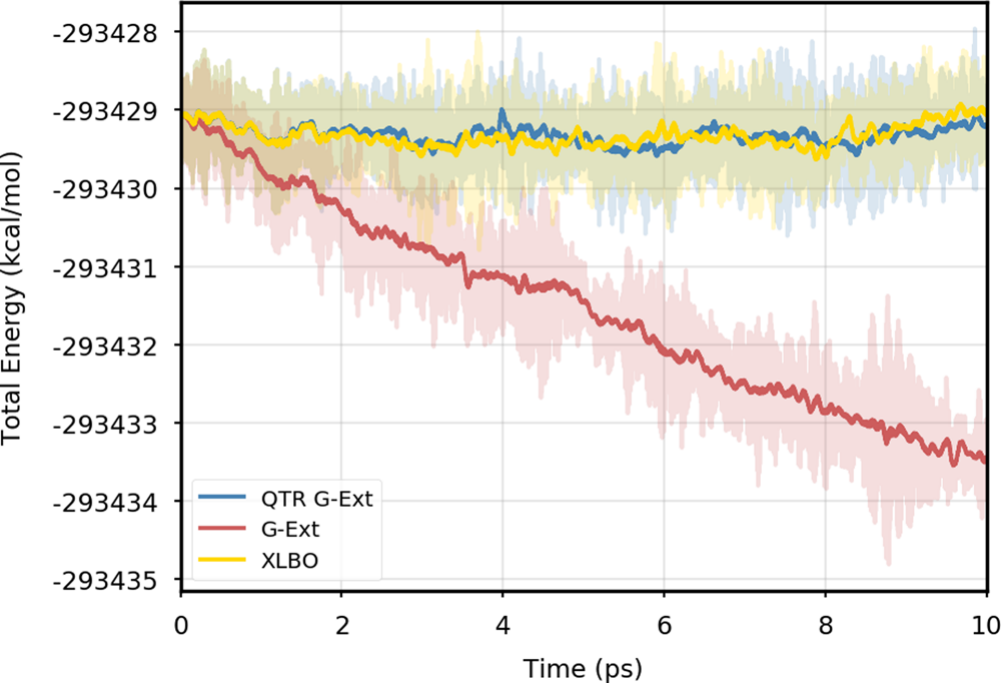
Total energy as a function of simulation time for DMABN using a 10^–5^ convergence threshold for the SCF.

**Figure 2:**
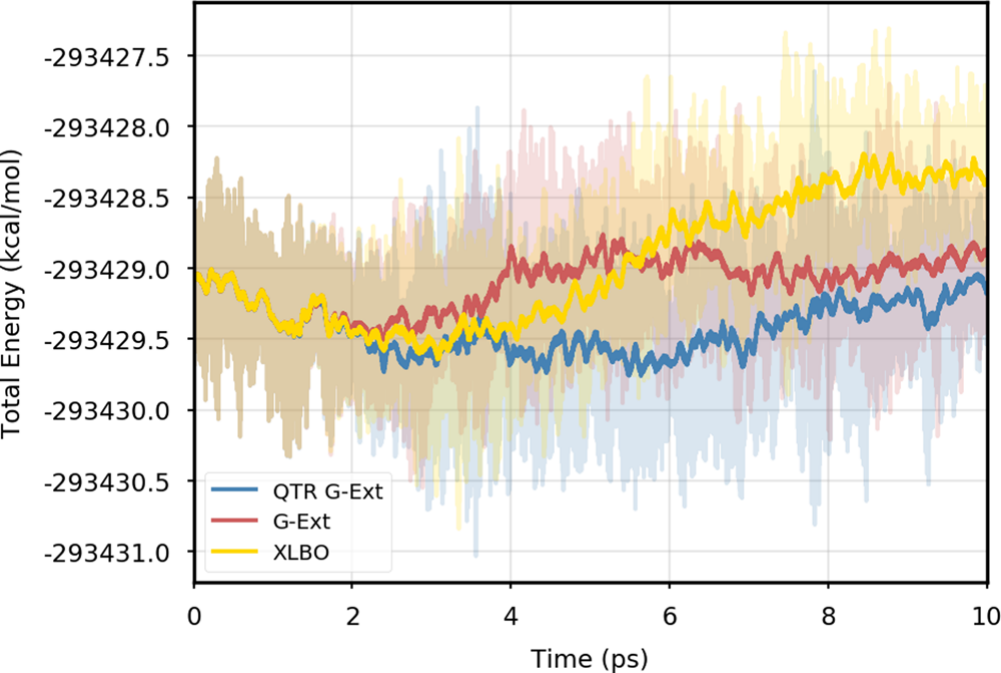
Total energy as a function of simulation time for DMABN using a convergence threshold for the SCF of 10^–7^.

**Table 2:** Short- and Long-Time Stability Analysis of the QTR G-Ext, G-Ext, and XLBO Methods^a^

	DMABN		3HF		AppA		OCP	
	STF	LTD	STF	LTD	STF	LTD	STF	LTD
QTR G-Ext	0.33	–0.01	0.62	–0.40	0.57	–0.08	0.36	–0.23
G-Ext	0.35	–0.43	0.61	–0.94	0.56	–0.93	0.38	–1.38
XLBO	0.32	0.01	0.57	–0.42	0.59	0.14	0.39	–0.28

a SCF convergence threshold 10^–5^.

**Table 3:** Short- and Long-Time Stability Analysis of the QTR G-Ext, G-Ext, and XLBO Methods^a^

	DMABN		3HF		AppA		OCP	
	STF	LTD	STF	LTD	STF	LTD	STF	LTD
QTR G-Ext	0.37	0.01	0.59	–0.30	0.53	0.18	0.38	–0.16
G-Ext	0.33	0.04	0.60	–0.27	0.54	0.06	0.38	–0.20
XLBO	0.32	0.13	0.64	–0.37	0.56	0.06	0.38	–0.08

a SCF convergence threshold 10^–7^.

The gain of our new methodology is not only in terms of accuracy (energy stability) but also in terms of the computational time of the simulation. Tables 4 and 5 report the average number of SCF iterations required to achieve convergence as well as the standard deviation for 10^–5^ and 10^–7^ SCF thresholds, respectively. We remark that each strategy requires *q* previous density matrices; before having them available, a standard SCF is performed. Therefore, for the computation of the average and standard deviation, we discard the first *q* points. The two tables show that for all the tested systems, the QTR G-Ext method requires the lowest number of SCF iterations for both convergence thresholds. Moving averages of SCF iteration numbers during the simulations for all systems and with both SCF convergence thresholds are reported in Section S2.

**Table 4:** Performance of the QTR G-Ext Method Compared to the G-Ext Method and XLBO Algorithm^a^

	DMABN		3HF		AppA		OCP	
	*k̅*	σ	*k̅*	σ	*k̅*	σ	*k̅*	σ
QTR G-Ext	3.04	0.22	2.98	0.21	3.00	0.02	2.96	0.31
G-Ext	3.55	0.85	3.16	0.69	3.03	0.54	2.91	0.41
XLBO	4.00	0.05	4.00	0.00	4.00	0.07	4.00	0.01

a Average *k̅* and standard deviation σ of the SCF iterations. Convergence threshold 10^–5^.

**Table 5:** Performance of the QTR G-Ext Method Compared with the G-Ext Method and XLBO Algorithm^a^

	DMABN		3HF		AppA		OCP	
	*k̅*	σ	*k̅*	σ	*k̅*	σ	*k̅*	σ
QTR G-Ext	5.42	0.69	5.42	0.80	5.37	0.84	4.86	0.83
G-Ext	7.33	0.63	6.96	0.79	6.56	0.75	5.83	0.87
XLBO	7.51	0.65	7.45	0.65	7.43	0.80	7.21	0.75

a Average *k̅* and standard deviation σ of SCF iterations. Convergence threshold 10^–7^.

The performances of the QTR G-Ext guess are also maintained for larger and smaller time steps. We compared QTR G-Ext and XLBO for time steps of 0.1, 0.25, 0.75, and 1 fs by running MD simulations for the DMABN system with an SCF convergence threshold of 10^–5^. All the results can be found in Section S3. Both methods show excellent energy conservation for the smaller time steps and afford reasonably stable simulations even for the larger ones, which is remarkable, as such simulations employing a time step that is too large to accurately sample molecular vibrations involving protons and are in general very noisy. For all time steps, QTR G-Ext requires a smaller average number of SCF iterations than XLBO. Finally, we tested the method for a looser SCF convergence of 10^–4^, again a value that should not be used for production applications, as the error on the SCF solution transfers to the forces, thus affecting the quality of the dynamics. The results are reported in Section S4. Again, good energy conservation is shown for both methods, and QTR G-Ext outperforms XLBO in terms of average number of SCF iterations required.

In conclusion, we presented the Quasi Time-Reversible Grassmann Extrapolation scheme, a new extrapolation method for ab initio molecular dynamics that not only allows for energy-conserving simulations but also exhibits overall excellent performances. Our numerical tests, performed on large, complex systems described with a polarizable multiscale strategy and taken from real-life production applications, show that QTR G-Etx is able to provide a guess density to BOMD simulations that allows the convergence of the SCF procedure in about 3 iterations on average for convergence thresholds that are typical of ground-state production runs, which is a 25% gain with respect to the state-of-the-art XLBO method. Tighter convergences, which are required for, e.g., time-dependent DFT excited-state simulations, can also be achieved in as little as 5–6 iterations. Furthermore, our numerical tests show that the new method does not introduce any significant bias in the guess density and thus exhibits very good energy conservation properties. This can be clearly seen by comparing the long-term drift observed in simulations for the two different SCF convergence thresholds used in our tests: while the previous G-Ext method shows a sharp increase in the drift going from 10^–7^ to 10^–5^ SCF convergence threshold, this is not the case for the QTR G-Ext method. We stress here that, due to the cost of BOMD simulations, every gain in performance is important, as it can easily translate into hundreds or thousands of saved CPU hours. The QTR G-Ext method is easy to implement and does not introduce any significant computational overhead and represents therefore an effective strategy to extend the applicability of BOMD simulations to larger and more complex systems.

## Supporting Information Available

Determination of the optimal parameters, plots of the total energy and number of SCF iterations along the dynamics for all tested systems, and results for the time step dependence and lower SCF convergence threshold (PDF)
