# Supplementary material for: A Quasi Time-Reversible Scheme Based on Density Matrix Extrapolation on the Grassmann Manifold for Born–Oppenheimer Molecular Dynamics
Source: J Phys Chem Lett. 2023 Oct 25;14(43):9720–6. doi: 10.1021/acs.jpclett.3c02098 (PMC10626629; doi:10.1021/acs.jpclett.3c02098)
Supplement: Supplementary file 1 — jz3c02098_si_001.pdf [file jz3c02098_si_001.pdf]

Supporting Information: A Quasi Time-Reversible scheme based on density matrix extrapolation on the Grassmann manifold for Born-Oppenheimer Molecular Dynamics

Federica Pes[1], Étienne Polack[2], Patrizia Mazzeo[1], Geneviève Dusson[3], Benjamin Stamm[4], and Filippo Lipparini[1,*]

[1]Dipartimento di Chimica e Chimica Industriale, Università di Pisa, Via G. Moruzzi 13, 56124 Pisa, Italy
[2]CERMICS, École des Ponts and Inria Paris, 6 & 8 avenue Blaise Pascal, 77455 Marne-la-Vallée, France
[3]Laboratoire de Mathématiques de Besançon, UMR CNRS 6623, Université de Franche-Comté, 16 route de Gray, 25030 Besançon, France
[4]Institute of Applied Analysis and Numerical Simulation, University of Stuttgart, 70569 Stuttgart, Germany
[*]filippo.lipparini@unipi.it

S1 Determination of optimal q and ε values

The parameters q and ε of the QTR G-Ext method were found by computing the error $\|\Gamma_n - \widetilde{\Gamma}_n\|$ and averaging it over the whole simulation for different values of q and ε, specifically $q = 3, 4, \ldots, 20$ and $\varepsilon = 0.001, 0.002, 0.005, 0.01, 0.02, 0.05$, and we selected the combination (q, ε) corresponding to the minimal error.

Table S1: Optimal q ed ε for each system and SCF tolerance.

	DMABN		3HF		AppA		OCP	
SCF tolerance	10^{-5}	10^{-7}	10^{-5}	10^{-7}	10^{-5}	10^{-7}	10^{-5}	10^{-7}
q	5	4	5	4	5	4	5	4
ε	0.005	0.001	0.005	0.001	0.005	0.002	0.005	0.002

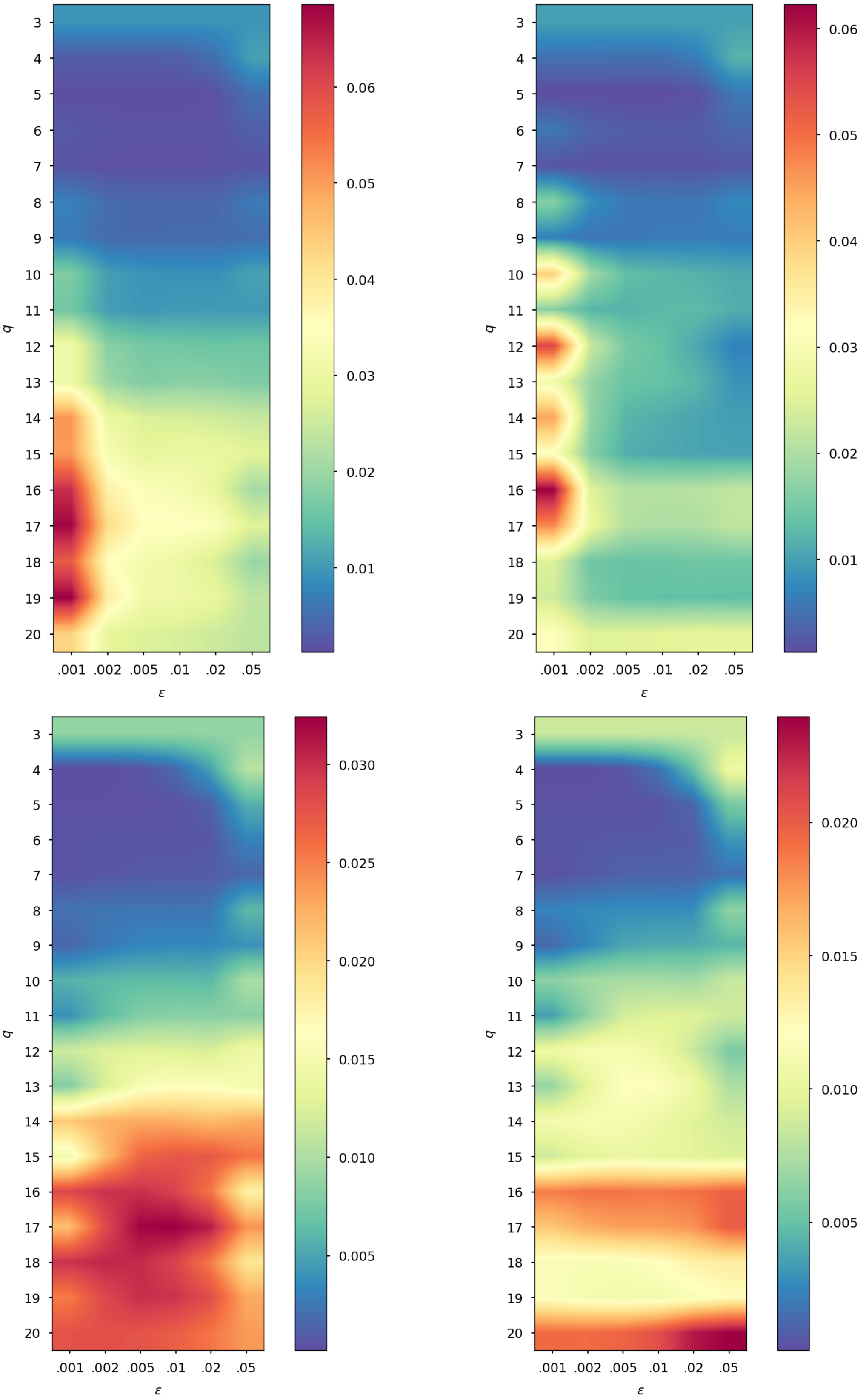

Figure S1: Error for DMABN (top) and for 3HF (bottom), with SCF convergence threshold of 10^{-5} (left panel) and 10^{-7} (right panel).

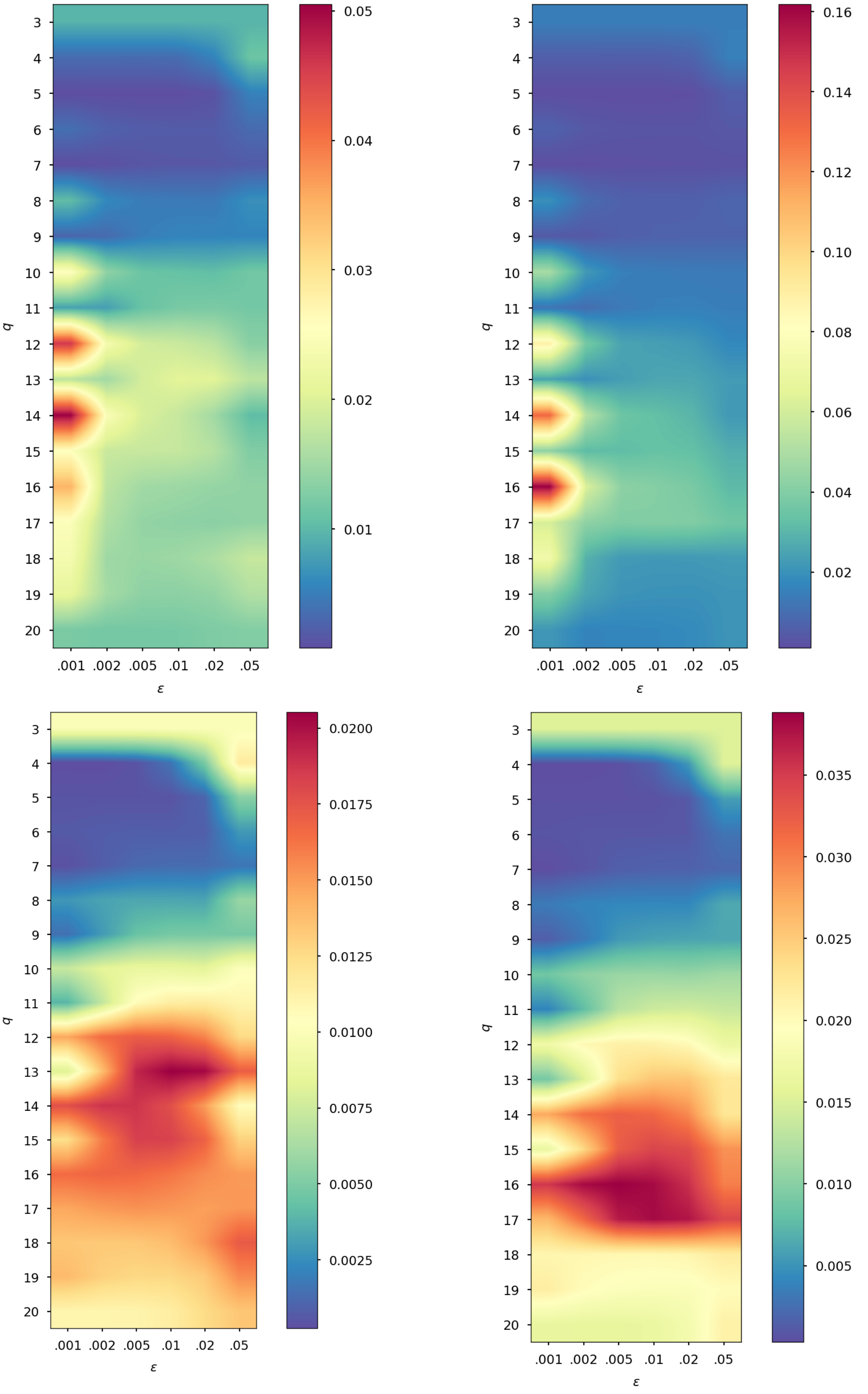

Figure S2: Error for AppA (top) and for OCP (bottom), with SCF convergence threshold of 10^{-5} (left panel) and 10^{-7} (right panel).

S2 Supplementary figures: Energy stability and number of SCF iterations

In the following, we report the total energy profile and number of SCF iterations per step for all the simulations performed in this work using the XLBO, G-Ext, QTR G-Ext, and TR schemes. The time-reversible scheme is obtained by computing the guess density as follows:

$$\widetilde{\Gamma}_n = -\widetilde{\Gamma}_{n-q} + \sum_{i=1}^{\widetilde{q}} \alpha_i \left(\Gamma_{n-i} + \Gamma_{n-q+i}\right), \tag{S1}$$

Eq. S1 is manifestly symmetric, and thus fully time-reversible. As mentioned in the main text, the fully TR scheme exhibits excellent stability, but poor performance, as the number of SCF iterations tends to quickly increase along the simulation, to the point that the extrapolation is not anymore beneficial.

S2.1 DMABN

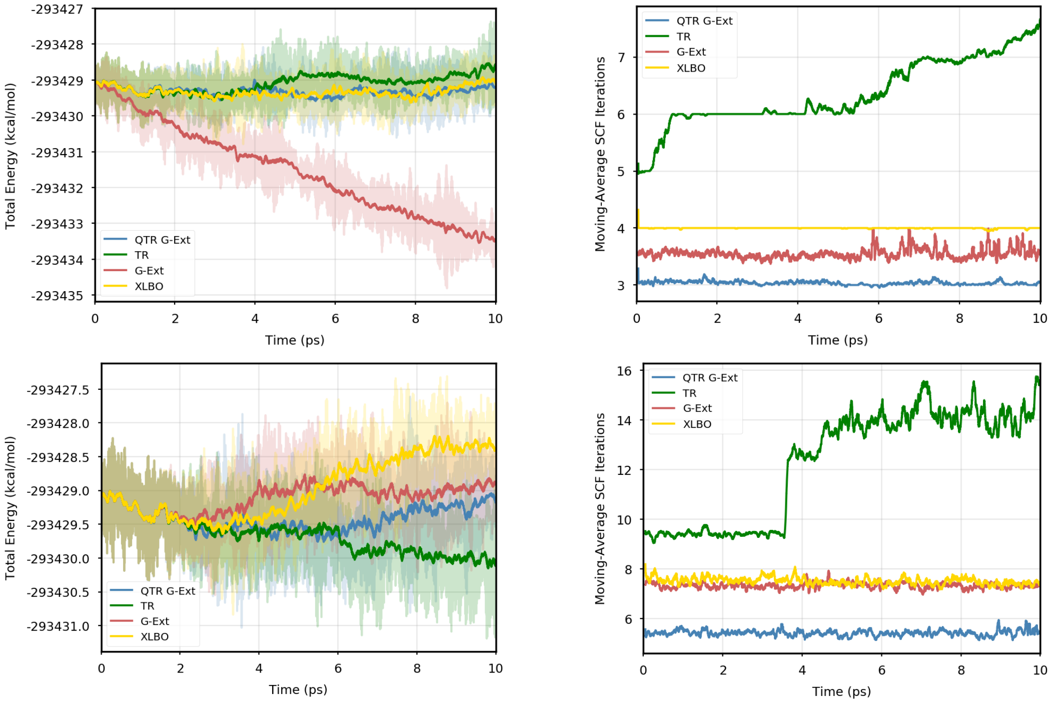

Figure S3: Comparison of total energies (left) and SCF iterations (right) along the simulation on DMABN with four extrapolation approaches: Quasi Time-Reversible (QTR G-Ext, blue line), Fully Time-Reversible (TR, green line), Grassmann Extrapolation (G-Ext, red line), and Extended Langrangian (XLBO, yellow line). For energy plots, soft colors are used to plot the actual energy values, whereas the marked line is the moving-average of the energy every 100 steps. The SCF iteration plots represent the moving-average of the number of iterations every 100 steps. SCF convergence threshold: 10^{-5} (top panel), 10^{-7} (bottom panel).

S2.2 3HF

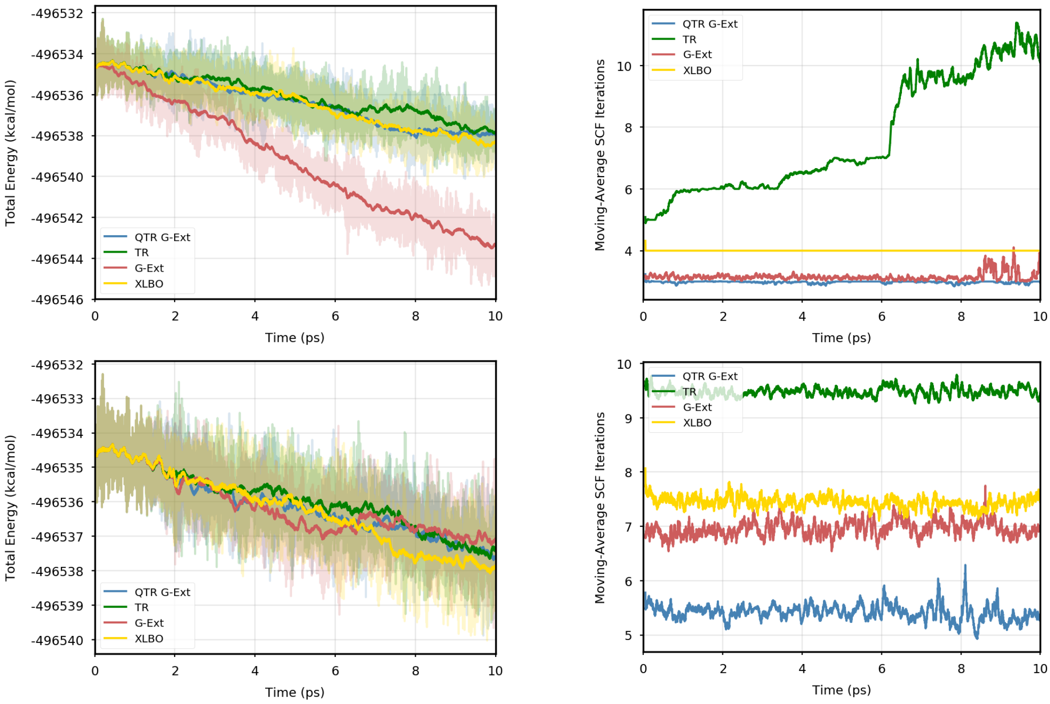

Figure S4: Comparison of total energies (left) and SCF iterations (right) along the simulation on 3HF with four extrapolation approaches: Quasi Time-Reversible (QTR G-Ext, blue line), Fully Time-Reversible (TR, green line), Grassmann Extrapolation (G-Ext, red line), and Extended Langrangian (XLBO, yellow line). For energy plots, soft colors are used to plot the actual energy values, whereas the marked line is the moving-average of the energy every 100 steps. The SCF iteration plots represent the moving-average of the number of iterations every 100 steps. SCF convergence threshold: 10^{-5} (top panel), 10^{-7} (bottom panel).

S2.3 AppA

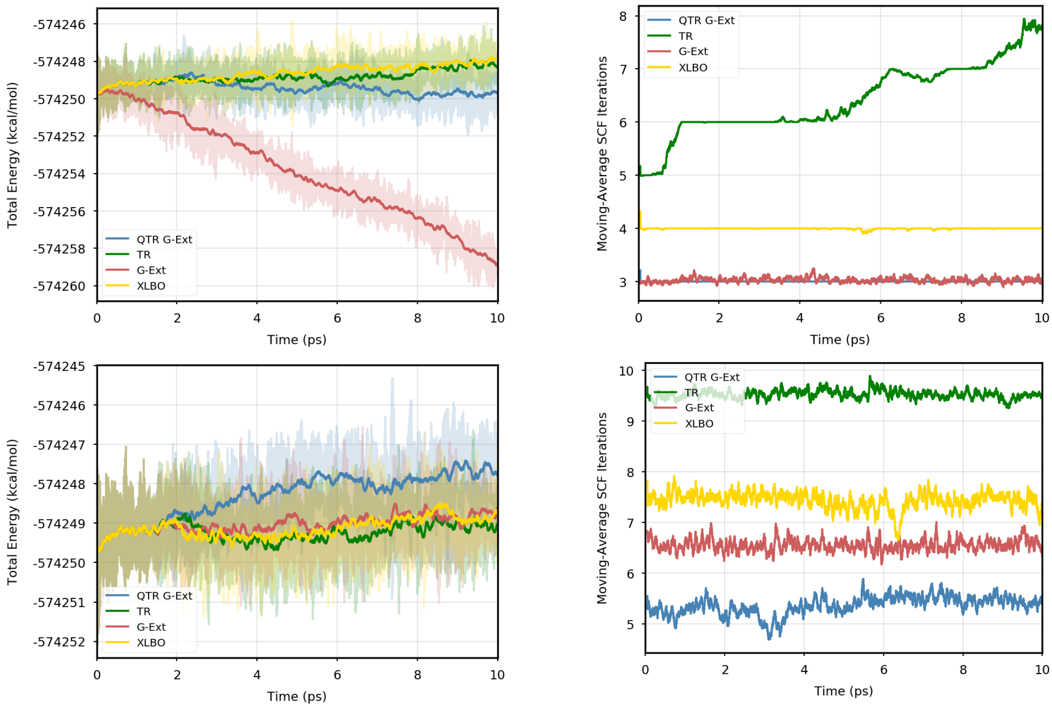

Figure S5: Comparison of total energies (left) and SCF iterations (right) along the simulation on AppA with four extrapolation approaches: Quasi Time-Reversible (QTR G-Ext, blue line), Fully Time-Reversible (TR, green line), Grassmann Extrapolation (G-Ext, red line), and Extended Langrangian (XLBO, yellow line). For energy plots, soft colors are used to plot the actual energy values, whereas the marked line is the moving-average of the energy every 100 steps. The SCF iteration plots represent the moving-average of the number of iterations every 100 steps. SCF convergence threshold: 10^{-5} (top panel), 10^{-7} (bottom panel).

S2.4 OCP

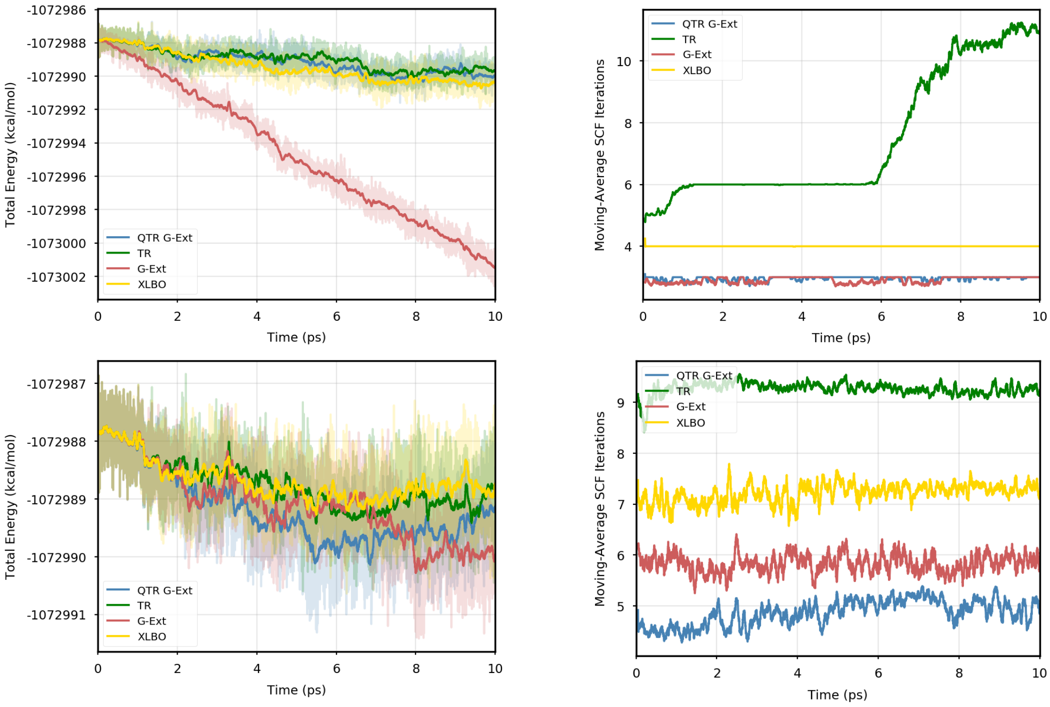

Figure S6: Comparison of total energies (left) and SCF iterations (right) along the simulation on OCP with four extrapolation approaches: Quasi Time-Reversible (QTR G-Ext, blue line), Fully Time-Reversible (TR, green line), Grassmann Extrapolation (G-Ext, red line), and Extended Langrangian (XLBO, yellow line). For energy plots, soft colors are used to plot the actual energy values, whereas the marked line is the moving-average of the energy every 100 steps. The SCF iteration plots represent the moving-average of the number of iterations every 100 steps. SCF convergence threshold: 10^{-5} (top panel), 10^{-7} (bottom panel).

S3 Supplementary tests: time step dependence

In the following, we report the results obtained for 20000 MD step on the system DMABN for QTR G-Ext and XLBO approaches using different time steps: 0.1, 0.25, 0.75, 1 fs. For completeness, the tables also show the results for time step equal to 0.5 fs. In these simulations, the SCF convergence threshold is 10^{-5}. For QTR G-Ext method, we estimate the optimal value of the parameters q and ε, as explained in Section S1:

Table S2: Optimal q ed ε for each time step.

time step (fs)	0.1	0.25	0.75	1
q	4	5	7	4
ε	0.01	0.002	0.001	0.01

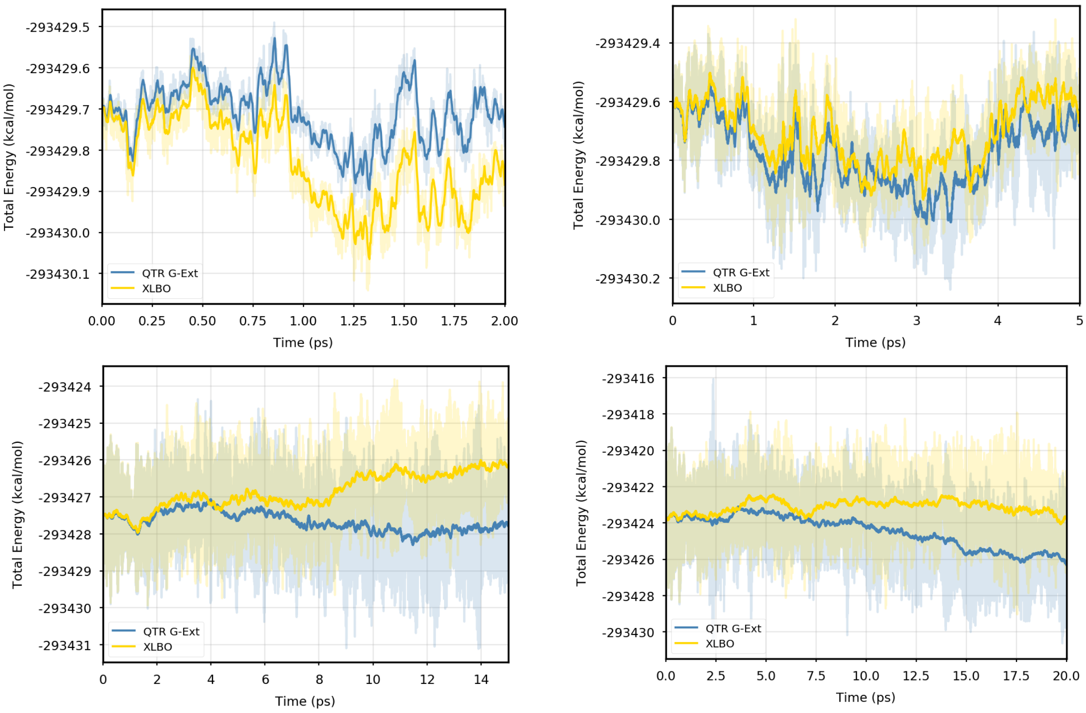

Figure S7: Comparison of total energies along the simulation on DMABN with two extrapolation approaches: Quasi Time-Reversible (QTR G-Ext, blue line) and Extended Langrangian (XLBO, yellow line). MD with different time steps: 0.1 fs (top-left), 0.25 fs (top-right), 0.75 fs (bottom-left), 1 fs (bottom-right). For energy plots, soft colors are used to plot the actual energy values, whereas the marked line is the moving-average of the energy every 100 steps. SCF convergence threshold: 10^{-5}.

Table S3: DMABN: Short- and Long-Time Stability Analysis of the QTR G-Ext and XLBO methods for molecular dynamics with different time steps. SCF convergence threshold 10^{-5}.

time step	0.1		0.25		0.5		0.75		1	
	STF	LTD	STF	LTD	STF	LTD	STF	LTD	STF	LTD
QTR G-Ext	0.02	-0.04	0.09	-0.01	0.33	-0.01	0.82	-0.04	1.39	-0.14
XLBO	0.02	-0.14	0.08	0.00	0.32	0.01	0.74	0.10	1.34	0.01

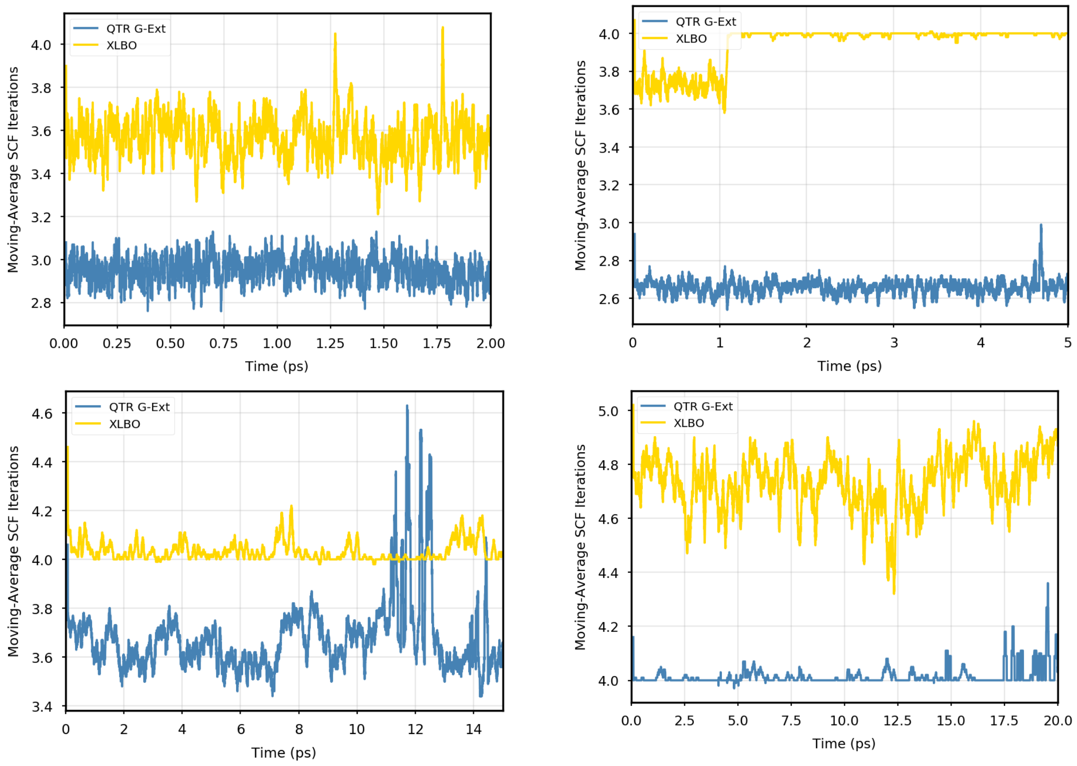

Figure S8: Comparison of SCF iterations along the simulation on DMABN with two extrapolation approaches: Quasi Time-Reversible (QTR G-Ext, blue line) and Extended Langrangian (XLBO, yellow line). MD with different time steps: 0.1 fs (top-left), 0.25 fs (top-right), 0.75 fs (bottom-left), 1 fs (bottom-right). The SCF iteration plots represent the moving-average of the number of iterations every 100 steps. SCF convergence threshold: 10^{-5}.

Table S4: DMABN: Performance of the QTR G-Ext method compared with the XLBO algorithm for molecular dynamics with different time steps. Average $\overline{k}$ and standard deviation σ of SCF iterations. SCF convergence threshold 10^{-5}.

time step	0.1		0.25		0.5		0.75		1	
	$\overline{k}$	σ	$\overline{k}$	σ	$\overline{k}$	σ	$\overline{k}$	σ	$\overline{k}$	σ
QTR G-Ext	2.95	0.65	2.66	0.81	3.04	0.22	3.68	0.61	4.02	0.20
XLBO	3.57	0.91	3.94	0.25	4.00	0.05	4.03	0.18	4.73	0.44

S4 Supplementary tests: lower SCF convergence threshold

In this section, we report the results obtained on the system DMABN for QTR G-Ext and XLBO approaches by establishing the convergence threshold to 10^{-4}. For QTR G-Ext method, the parameters are $q = 5$ and $\varepsilon = 0.005$. For completeness, the tables also show the results for convergence threshold equal to 10^{-5} and 10^{-7}. We performed 10 ps BOMD simulations, with 0.5 fs time step.

Table S5: DMABN: Short- and Long-Time Stability Analysis of the QTR G-Ext and XLBO methods for molecular dynamics with different SCF convergence thresholds.

conv. threshold	10^{-4}		10^{-5}		10^{-7}	
	STF	LTD	STF	LTD	STF	LTD
QTR G-Ext	0.37	-0.27	0.33	-0.01	0.37	0.01
XLBO	0.45	-0.00	0.32	0.01	0.32	0.13

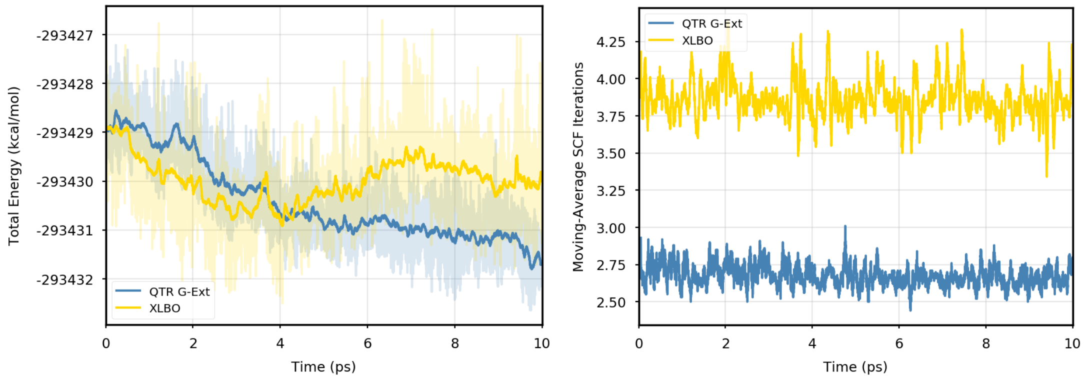

Figure S9: Comparison of total energies (left) and SCF iterations (right) along the simulation on DMABN with two extrapolation approaches: Quasi Time-Reversible (QTR G-Ext, blue line) and Extended Langrangian (XLBO, yellow line). For energy plots, soft colors are used to plot the actual energy values, whereas the marked line is the moving-average of the energy every 100 steps. The SCF iteration plots represent the moving-average of the number of iterations every 100 steps. SCF convergence threshold: 10^{-4}.

Table S6: DMABN: Performance of the QTR G-Ext method compared with the XLBO algorithm for molecular dynamics with different SCF convergence thresholds. Average $\overline{k}$ and standard deviation σ of SCF iterations.

conv. threshold	10^{-4}		10^{-5}		10^{-7}	
	$\overline{k}$	σ	$\overline{k}$	σ	$\overline{k}$	σ
QTR G-Ext	2.68	0.97	3.04	0.22	5.42	0.69
XLBO	3.86	0.75	4.00	0.05	7.51	0.65
